# Fast dynamics in the HPA axis: Insight from mathematical and experimental studies

**DOI:** 10.1016/j.coemr.2022.100403

**Published:** 2022-12

**Authors:** Jamie J. Walker, Nicola Romanò

**Affiliations:** 1 Department of Mathematics and Statistics, Faculty of Environment, Science and Economy, University of Exeter, UK; 2 Henry Wellcome Laboratories for Integrative Neuroscience and Endocrinology, University of Bristol, UK; 3 Centre for Discovery Brain Sciences, University of Edinburgh, UK

**Keywords:** HPA axis, Oscillation, Ultradian, Corticotroph, CRH, AVP, ACTH, Glucocorticoid, Modelling, Stress

## Abstract

The activity of the hypothalamic-pituitary-adrenal (HPA) axis is characterised by complex dynamics spanning several timescales. This ranges from slow circadian rhythms in blood hormone concentration to faster ultradian pulses of hormone secretion and even more rapid oscillations in electrical and calcium activity in neuroendocrine cells of the hypothalamus and pituitary gland. Here, we focus on the system's oscillations on the short timescale. We highlight some of the mathematical modelling and experimental work that has been carried out to characterise the mechanisms regulating this highly dynamic mode of neuroendocrine signalling and discuss some future directions that may be explored to enhance understanding of HPA function.

## Highlights

• Fast dynamics in HPA axis activity is important for physiological function.
• Coupling mathematical modelling and experimental approaches can shed light on the mechanisms underlying these dynamics.
• Recent computational and experimental advances are enabling a deeper understanding of the system at multiple scales.

## Introduction

Oscillatory dynamics underlies neuroendocrine signalling and control. The stress-responsive hypothalamic-pituitary-adrenal (HPA) axis is a good example of this, where the pulsatile release of vital glucocorticoid hormones (principally cortisol in man and corticosterone in rodents) from the adrenal glands results in an ultradian oscillation in blood glucocorticoid concentration. The mean period of these oscillations varies between species, and is typically approximately hourly in rodents [1], and 1–3 h in humans [2]. The nature of this ultradian activity depends on many factors, including genetic and epigenetic status as well as (patho)physiological state [3].

Central control of glucocorticoid secretion is mediated by neurons in the hypothalamic paraventricular nucleus (PVN) expressing corticotrophin-releasing hormone (CRH) and arginine vasopressin (AVP). These neuropeptides are released into the hypophyseal portal vessels at the median eminence, thus stimulating adrenocorticotrophic hormone (ACTH) secretion from anterior pituitary corticotroph cells. In turn, ACTH stimulates the synthesis and secretion of glucocorticoids from the adrenal cortex. Glucocorticoids exert their influence over a wide range of timescales, and these effects are mediated by both the glucocorticoid receptor (GR) and mineralocorticoid receptor (MR) [4]. Glucocorticoids inhibit their own secretion in a classic neuroendocrine negative feedback loop by acting back on the hypothalamus and anterior pituitary to inhibit the secretion of CRH, AVP, and ACTH [5].

Microdialysis studies have shown that the ultradian glucocorticoid oscillation in the blood is paralleled within brain tissue [6,7]. Several studies have highlighted the physiological significance of these oscillations. In particular, glucocorticoid oscillations induce bursts of GR binding to DNA and subsequent pulsatile dynamics in GR-dependent gene expression [8∗, 9, 10]. In addition to dynamically regulating genomic signalling, glucocorticoids also affect neuronal activity even more rapidly (i.e., within minutes) through non-genomic actions; that is, independently of gene expression and de novo synthesis of mRNA and protein [11]. These fast effects may well underlie the acute behavioural responses that have been observed within minutes of glucocorticoid exposure [12]. The phase of the ultradian oscillation itself is important in regulating both hormonal [1] and behavioural [13] responses to acute stress, which are most significant when the stress coincides with the rising phase of the oscillation.

The amplitude of the ultradian glucocorticoid rhythm is modulated in a circadian manner by the hypothalamic suprachiasmatic nucleus (SCN) through direct and indirect connections to the PVN, which regulate CRH gene expression and CRH secretion. The sensitivity of the adrenal cortex to ACTH is also under circadian control with sensitivity peaking during the active phase; an effect that appears to be dependent on a direct SCN polysynaptic neuronal connection to the adrenal via the autonomic nervous system [14] as well as an endogenous intra-adrenal circadian clock mechanism [15]. Loss of SCN function abolishes the circadian, but not ultradian, glucocorticoid rhythm, highlighting the pivotal role of the SCN in regulating circadian HPA activity [16]; thus, understanding circadian regulation of HPA activity primarily amounts to understanding the function of the SCN and its neural regulation of the PVN and adrenal glands, which is beyond the scope of this article and has been reviewed comprehensively elsewhere [17,18]. Here, we highlight some of the experimental and mathematical work that has been carried out to understand the regulation of HPA oscillations at the faster ultradian timescale and discuss some future avenues for research in this area.

## Ultradian pulsatility in the system

The question of how the HPA axis generates ultradian pulsatile dynamics in hormone secretion has remained a puzzle ever since episodic cortisol secretion was observed in man over half a century ago [19]. Ultradian pulsatile patterns of ACTH were also later observed [20], and these have been shown to be tightly correlated with the pulsatile glucocorticoid pattern [2].

Neural signalling to the anterior pituitary is encoded in the dynamic patterns of hypothalamic peptides released into the hypophyseal portal vessels. This has led to the basic hypothesis that there exists a neural oscillator generating pulsatile bursts of CRH/AVP secretion, in turn driving pulsatile secretion of ACTH and glucocorticoids; a concept that bears strong similarity to the tightly correlated pulsatile secretion of gonadotrophin-releasing hormone and luteinizing hormone [21]. Indeed, pulsatile patterns of CRH and/or AVP secretion have been observed at the median eminence of the rat [22] and in the hypophyseal-portal circulation of the sheep [23]. In light of these data, mathematical models of the HPA axis have been developed that incorporate pulsatile hypothalamic activity, and these have been analysed to understand how hypothalamic-driven pulsatility in the system is regulated by glucocorticoid feedback acting over multiple sites and time domains [24,25].

To date, there has been relatively little biological description of the neural oscillator mechanism(s) involved in controlling PVN activity and the pulsatile secretion of CRH and AVP, and this is perhaps the main reason for the lack of modelling impetus in this area. Ex vivo studies demonstrating episodic CRH release from cultured hypothalamic explants from the macaque suggest that an oscillator mechanism resides within the hypothalamus [26]. Ultradian bursts of multi-unit neuronal activity have been observed within the SCN of the hamster [27], but ultradian HPA activity is maintained following SCN loss-of-function [16]. Within the PVN itself, CRH-dependent microcircuits of excitatory and inhibitory feedback connections have begun to be characterised [28], and as experimental investigations continue to shed light on the regulatory mechanisms within this neuronal population, mathematical investigation into their potential role in generating and regulating CRH/AVP pulsatility will hopefully follow.

Another hypothesis, which has been proposed and explored primarily through mathematical modelling, has been that the negative feedback action of glucocorticoids on the system can cause it to become dynamically unstable, giving rise to ultradian oscillations in secretory activity. In this scenario, the system as a whole would function as a pulse generator. Ultradian oscillations have been explored through systems of differential equations in which variables describe the overall secretory activity of an entire cell population; mathematical analysis of these “black box” models of the HPA axis has shown that an ultradian oscillation can indeed arise from negative glucocorticoid feedback in the system. Most models have considered negative glucocorticoid feedback at the level of both the hypothalamus and the anterior pituitary, and it is this dual-feedback mechanism that generates the system-level ultradian oscillation [29].

A key feature of dual-feedback models is that the CRH, ACTH and glucocorticoid oscillations are generated at the same frequency. However, some experimental studies have found a lack of tight concordance between basal CRH/AVP secretion and ACTH and glucocorticoid pulsatility [23], as well as a lack of sensitivity of CRH pulsatility to glucocorticoid withdrawal or dexamethasone treatment [26,30]. This suggests that hypothalamic pulsatility may be sustained independently of glucocorticoid feedback, and that ultradian activity in the pituitary-adrenal system may also be regulated by mechanisms independent from hypothalamic control. This concept has been explored in principle using a qualitative mathematical model of the pituitary-adrenal system incorporating glucocorticoid feedback at the pituitary mediated via GR and driving it with a constant pattern of CRH/AVP [31]. Numerical analysis of this model has suggested that the feedforward–feedback interaction between the pituitary and adrenal can support endogenous ultradian oscillations at a physiological frequency. These theoretical predictions have subsequently been confirmed in vivo in the rat [32], lending further support to the existence of a sub-hypothalamic pituitary-adrenal oscillator. It remains to be fully established how hypothalamic pulsatility and the pituitary-adrenal oscillator interact to control physiological patterns of glucocorticoids.

## Electrical oscillations in corticotroph cells

Work suggesting that feedforward and feedback interactions between the pituitary and adrenal can give rise to ultradian oscillations in the system implies that the corticotroph cells of the pituitary play a central role in controlling the dynamics of the system: these cells must integrate the dynamic output from the hypothalamus and the oscillatory activity generated within the pituitary-adrenal system. Mathematical models of corticotroph activity have focused on describing the mechanisms of action potential firing, primarily through the Hodgkin-Huxley formalism. In particular, systems of ordinary differential equations describing several ionic currents present in these cells have been used to describe their spontaneous and secretagogue-induced electrical activity.

Action potentials in corticotrophs are calcium-dependent and associated with rises in intracellular calcium concentration [33]. Early models [34,35] include a central role for L-type calcium currents (I_Ca-L_) and to a lesser extent for T-type, in the generation of action potentials. While these models mainly focus on the central role of calcium currents, they also account for potassium currents. These include the delayed-rectifier potassium channels (I_K-dr_) and the voltage and calcium-activated (I_K_–_Ca_) large conductance potassium (BK) and small conductance potassium (SK) channels.

The role of potassium channels has been explored in detail in later models, specifically for analysing secretagogue-induced firing [36,37]. These models explore the role of CRH, AVP and glucocorticoids in shaping the electrical activity of corticotrophs. At rest, corticotrophs display heterogeneous firing behaviour; most corticotrophs fire low-frequency single action potentials, while some display higher frequency firing and ‘pseudo-plateau bursting’ [36,38]. CRH increases firing frequency through protein kinase A (PKA)-mediated phosphorylation of L-type channels. This increase in frequency is accompanied by a switch in firing from single spikes to pseudo plateau bursting, a pattern thought to be more efficient for the secretion of hormones from pituitary cells [39].

BK channels play a central role in mediating the spiking-to-bursting switch. CRH reduces BK currents in a PKA-dependent manner [36,40]; pretreatment with glucocorticoids can inhibit this transition, as well as spontaneous activity, through BK-dependent and -independent mechanisms [41]. Modelling efforts focussed on the role of BK have theorised the presence of two sub-populations of BK channels (BK_near_ and BK_far_) distinguished by their spatial association with L-type calcium channels [36]. It has been assumed that BK_near_ channels represent stress-regulated exon (STREX)-type channels, while BK-far channels represent ZERO-type BK channels lacking the STREX exon. There is currently no experimental proof of the physical association of these variants with calcium channels in corticotrophs; however, recent modelling and experimental results indicate that an increase in the association between BK and calcium channels and a change in their activation constant could underlie the changes in firing activity recorded in chronic stress [42].

Contrary to CRH, stimulation with AVP only increases firing frequency without changing the firing pattern [36], although some reports show AVP-induced bursting [43]. Whether these differences are methodological or reflect a prominent heterogeneity in corticotroph behaviour has not been determined.

## Future directions

We have highlighted some of the modelling and experimental approaches that have been used to investigate the fast and complex dynamics of the HPA axis. These models have helped to deepen our understanding of the system's behaviour, yet many questions remain. Here, we identify some of the challenges that we believe can be addressed through a combination of experimental and modelling approaches informing each other.

It is the case for most models that complexity comes at the cost of scale; system-wide models consider each of the compartments of the axis as “black boxes”, while models exploring the role of molecular components are generally limited to single cells. Recent improvements in computing power should allow linking these spatial scales and defining system-wide models consisting of individually modelled cells. Such multiscale models may allow exploration of the impact of changes of single cell function on system-level dynamics. In addition, they may enable understanding of the role of cellular heterogeneity in the HPA system, which has been experimentally described at the level of electrical activity [38], calcium responses to CRH and AVP [43,44], and transcriptional activity as highlighted by recent single-cell RNA sequencing of HPA axis components [45].

The generation of multiscale models will require a deeper biological understanding of the molecular processes at the level of single corticotrophs, such as the intracellular pathways and cellular dynamics controlling the secretion of ACTH from vesicles. Understanding how corticotrophs communicate with each other and with other pituitary cell types will enable linking single-cell function to tissue-level population activity. Functional cellular networks have been described for other cell types in the pituitary [46], but it remains to be demonstrated whether the distinctive anatomical corticotroph network holds physiological significance [47].

Beyond understanding the “basal” dynamic function of the HPA axis, mathematical models of this system can be adapted to explain how its dynamic activity changes depending on the physiological or environmental context. This has been explored for conditions such as acute psychological stress [48] and inflammatory stress [49,50]. Expanding models of the HPA axis to include SCN circadian regulation enables the characterisation of environmental effects such as a disrupted light environment [51], and coupling models of the HPA axis with models of other neuroendocrine systems, such as the hypothalamic–pituitary–gonadal axis [52], can offer a mechanistic understanding of the complex dynamic interplay between them.

## Funding

JJW acknowledges financial support from the 10.13039/501100000265Medical Research Council (MR/N008936/1). NR acknowledges financial support from the Medical Research Council (MR/V012290/1).

## Conflict of interest statement

Nothing declared.
